# Association of a rare NOTCH4 coding variant with systemic sclerosis: a family-based whole exome sequencing study

**DOI:** 10.1186/s12891-016-1320-4

**Published:** 2016-11-09

**Authors:** Christopher J. Cardinale, Dong Li, Lifeng Tian, John J. Connolly, Michael E. March, Cuiping Hou, Fengxiang Wang, James Snyder, Cecilia E. Kim, Rosetta M. Chiavacci, Patrick M. Sleiman, Jon M. Burnham, Hakon Hakonarson

**Affiliations:** 1 Center for Applied Genomics, Abramson Pediatric Research Center, The Children’s Hospital of Philadelphia, 3615 Civic Center Blvd Ste 1216, Philadelphia, PA 19104 USA; 2 Department of Pediatrics, Perelman School of Medicine, University of Pennsylvania, Philadelphia, PA 19104 USA; 3 Division of Rheumatology, Department of Pediatrics, The Children’s Hospital of Philadelphia, Philadelphia, PA 19104 USA

**Keywords:** Whole exome sequencing, Systemic sclerosis, Scleroderma, NOTCH4, Mendelian genetics

## Abstract

**Background:**

Systemic sclerosis (SSc) is a rheumatologic disease with a multifactorial etiology. Genome-wide association studies imply a polygenic, complex mode of inheritance with contributions from variation at the human leukocyte antigen locus and non-coding variation at a locus on chromosome 6p21, among other modestly impactful loci. Here we describe an 8-year-old female proband presenting with diffuse cutaneous SSc/scleroderma and a family history of SSc in a grandfather and maternal aunt.

**Methods:**

We employed whole exome sequencing (WES) of three members of this family. We examined rare missense, nonsense, splice-altering, and coding indels matching an autosomal dominant inheritance model. We selected one missense variant for Sanger sequencing confirmation based on its predicted impact on gene function and location in a known SSc genetic locus.

**Results:**

Bioinformatic analysis found eight candidate variants meeting our criteria. We identified a very rare missense variant in the regulatory NODP domain of *NOTCH4* located at the 6p21 locus, c.4245G > A:p.Met1415Ile, segregating with the phenotype. This allele has a frequency of 1.83 × 10^−5^ by the data of the Exome Aggregation Consortium.

**Conclusion:**

This family suggests a novel mechanism of SSc pathogenesis in which a rare and penetrant coding variation can substantially elevate disease risk in contrast to the more modest non-coding variation typically found at this locus. These results suggest that modulation of the *NOTCH4* gene might be responsible for the association signal at chromosome 6p21 in SSc.

**Electronic supplementary material:**

The online version of this article (doi:10.1186/s12891-016-1320-4) contains supplementary material, which is available to authorized users.

## Background

Systemic sclerosis, also known as SSc or scleroderma, is an autoimmune disease characterized by a triad of microvascular dysfunction, immune dysfunction, and generalized fibrosis in connective tissues and organs [1]. One of the most concerning aspects of the disease is that mortality has not improved greatly over the last several decades because there is a critical lack of therapies to address the fibrotic process [2]. The urgent need for innovation in SSc is one of the motivations of the genetics community in attempting to explore the hereditary underpinnings of this condition. Genetic epidemiology has shown convincing evidence of familial aggregation, with increased risk to siblings and first degree relatives as well as substantial epidemiologic overlap with other autoimmune diseases [3]. The etiology of the disease is multifactorial, with poorly-understood environmental influences and a complex mode of genetic inheritance. Since SSc is a relatively rare disease, most cases appear sporadically, without family history [3]. Recent advances in genomic technology, such as high-density genotyping on microarrays, have made possible genome-wide association studies (GWAS) that have enhanced the genetic understanding of SSc.

The single stand-out genetic risk for SSc is associated with an array of variants in the major histocompatibility complex (MHC), containing the human leukocyte antigen (HLA) genes [4], a pattern seen in a wide array of autoimmune diseases. The first large GWAS revealed associations with non-coding SNPs at a number of loci in addition to the HLA, including *IRF5*, *STAT4*, *CD247*, *CDH7*, and *IRF4* [4]. Later GWAS on specific biomarkers and clinical phenotypes [5] as well as high-density genotyping in selected regions on the Immunochip [6] have yielded additional associations. A recent study used whole exome sequencing (WES) in a modest number of cases to identify specifically protein-altering variants, revealing a low-frequency variant in *ATP8B4* which was enriched among SSc cases compared to controls (odds ratio = 6.1) [7].

Of particular interest is an association from GWAS with the *NOTCH4* locus which lies on chromosome 6p21 in proximity to the HLA region. This locus gave an association with the presence of anti-centromere antibody (ACA) or anti-topoisomerase I antibody (ATA) in SSc with *P* < 8.84 × 10^−21^, OR = 0.55 which were independent of the HLA class II associations [5]. The *NOTCH4* locus has previously been associated, independently from the HLA, with other autoimmune disorders including ulcerative colitis [8], rheumatoid arthritis [9], and alopecia areata [10] and age-related macular degeneration [11].

**Fig. 1:**
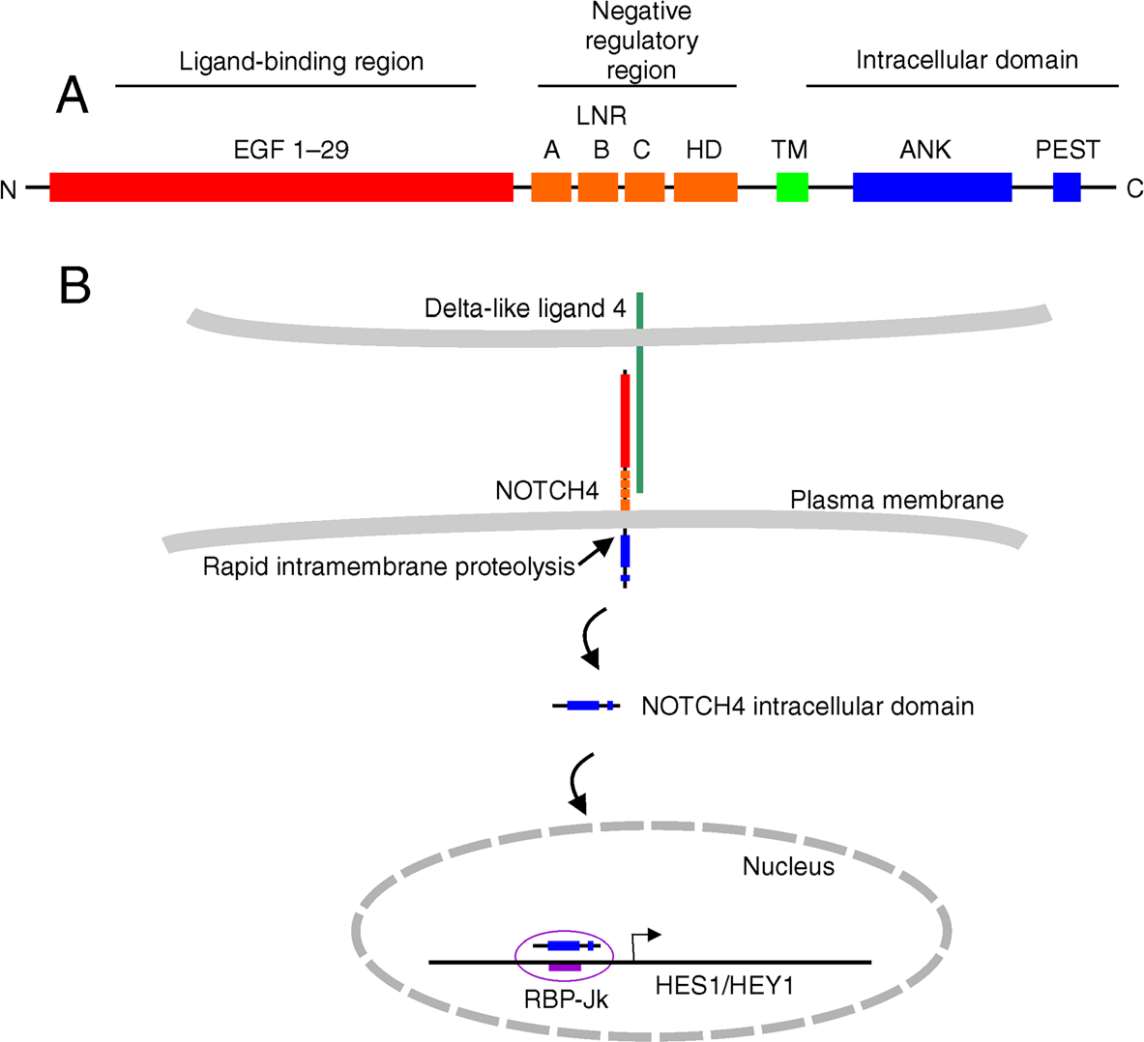
An overview of proposed NOTCH4 structure and signaling. **a** The receptor NOTCH4 is a 2002-amino acid transmembrane protein with its N terminus situated extracellularly (Type I membrane protein). From N terminus to C terminus it consists of 36 epidermal growth factor (EGF) repeats; 3 NOTCH domains (LNR, Lin-12/Notch repeat); a heterodimerization domain (HD) containing the NODP domain; a transmembrane domain (TM); and an intracellular domain. The intracellular domain is made up of ankyrin repeats (ANK) and a PEST domain. **b** The mechanism of NOTCH family signaling involves binding by a ligand of the DSL family (e.g., DLL4), which results in cleavage of the intracellular domain at the plasma membrane. The intracellular domain translocates to the nucleus where it binds DNA-binding transcription factor RBP-Jk and acts as a transcriptional activator of effector gene transcription such as *HES1* and *HEY1*

NOTCH4 is a member of a four-gene family (NOTCH1 to 4) and is expressed specifically in endothelial cells [12]. NOTCH proteins are transmembrane receptors activated by transmembrane ligands of the DSL family (Delta/Serrate/Lag-2). Based on structural investigation of the well-studied NOTCH1 family member, binding of the ligand triggers a conformational change in the negative regulatory region (NRR), consisting of LNR repeats and a heterodimerization (HD) region consisting of a NOD and a NODP domain (NOTCH domain) [13, 14]. The isomerization of the NRR unmasks protease cleavage sites, which leads to the intracellular domain of the NOTCH1 receptor being cleaved off. The free intracellular domain translocates to the nucleus and binds to the DNA-binding transcription factor RBP-Jk, activating transcription (Fig. 1).

There are multiple phenotypic manifestations caused by the activation of NOTCH4 in a mouse model system. Ectopic overexpression of the free NOTCH4 intracellular domain in mammary epithelium leads to oncogenic transformation and mammary carcinogenesis [14, 15]. Expression of the free intracellular domain in vascular endothelium is embryonic lethal, with disorganized vascular networks, fewer small vessels, and compromised vessel-wall integrity, demonstrating an important role for NOTCH4 signaling in the development of the vascular system [16]. The role of NOTCH4 in vascular development has significant implications for SSc because the pathological process is thought to be driven by damage to the microvasculature caused by dysfunctional endothelial cells. Morphological changes and activation of endothelial cells are often the earliest detectable sign of disease [17]. This vascular damage leads to reduction in the number of small vessels, thickening of the vessel wall, and luminal narrowing, eventually leading to tissue hypoxia [17]. The connection between vasculopathy and fibrosis is unclear but is under investigation.

Here we describe a family presenting with a three-generation history of SSc in an apparently autosomal-dominant mode of inheritance. We used whole exome sequencing to identify rare mutations which segregate as expected in the pedigree and which might be contributory to the development of the disease. Our characterization of a very rare missense variant in the NOTCH4 NODP domain is described below. The NODP domain is of particular interest because in the homologous NOTCH1 receptor, mutations in this domain result in constitutive activation and consequent T cell acute lymphoblastic leukemia [18].

## Methods

### Whole exome sequence analysis

The SSc phenotype of the proband was determined by a senior pediatric rheumatologist and family history was confirmed.

After written informed consent was obtained, genomic DNA was extracted from the peripheral blood lymphocytes of the proband, mother, affected maternal aunt, unaffected maternal uncle and unaffected maternal grandmother. Whole exome capture was carried out for the two patients and unaffected maternal grandmother using the SureSelect Human All Exon version 3 kit (Agilent Technologies, Santa Clara, CA), according to the manufacturer’s protocols. Sequencing was carried out on the HiSeq 2000 instrument (Illumina, San Diego, CA) using the manufacturer’s recommended procedure. Mapping of next generation sequencing reads and variant calling was performed with the Burrows-Wheeler aligner (BWA) [19] and the variants called using the Genome Analysis Toolkit (GATK) [20]. The results were filtered to exclude synonymous variants, variants with minor allele frequency greater than 0.5 % under an autosomal dominant model, and variants previously identified in controls by our in-house exome variant database using ANNOVAR [21]. ANNOVAR produced the data in Additional file 1: Table S1, including functional impact scores (SIFT [22], PolyPhen2 [23], and GERP [24]). The kinship coefficient was calculated between every two samples via KING to confirm reported relationships [25]. Co-segregation patterns were confirmed by Sanger sequencing in 5 members whose DNA was available using standard PCR amplicons.

## Results

### Clinical history

**Fig. 2:**
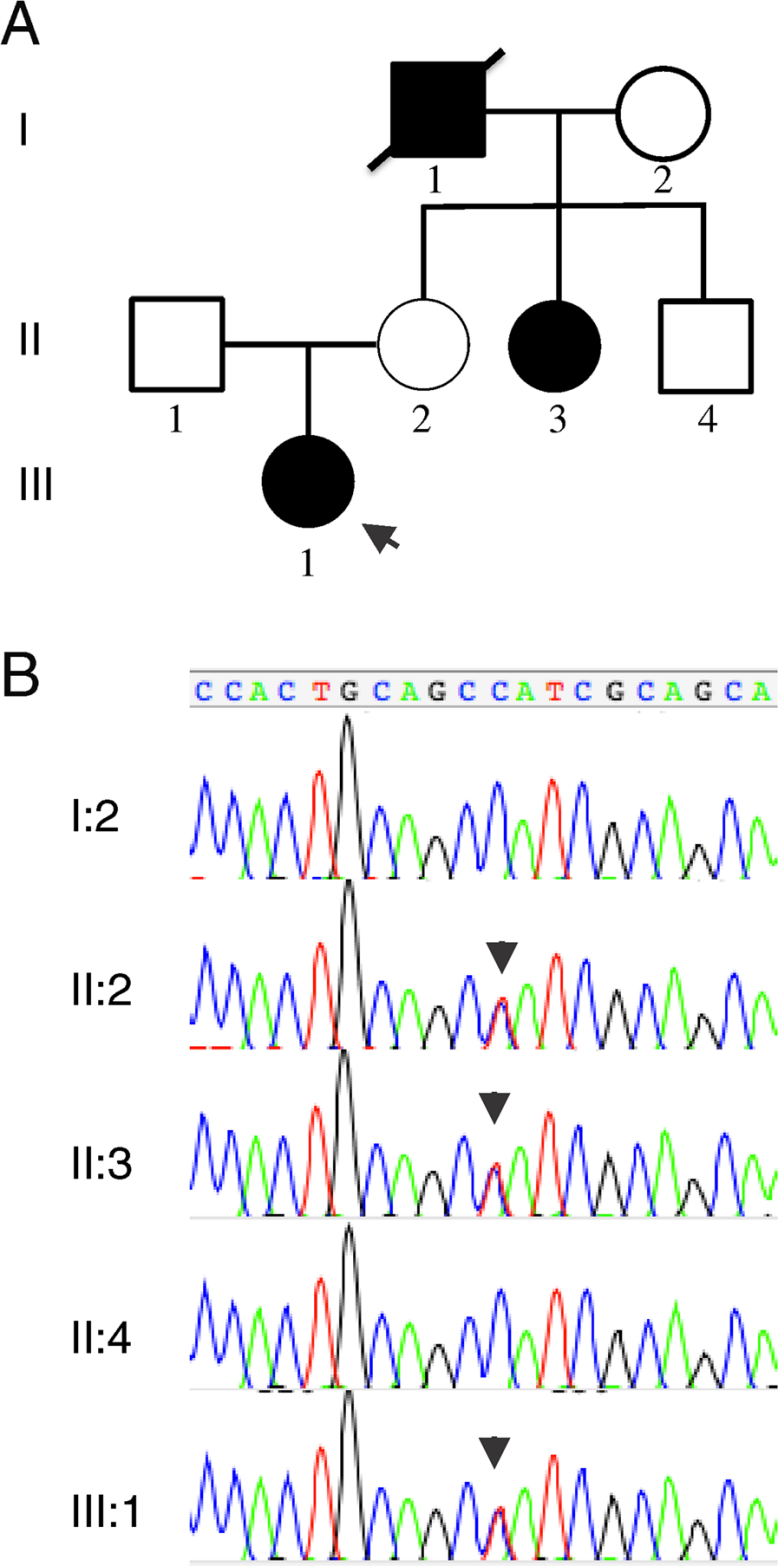
A family with systemic sclerosis segregating a mutation in NOTCH4. **a** Pedigree of the family under study. There are three affecteds (filled symbols). Proband is indicated by an arrow. **b** Sanger sequencing validation of the NOTCH4 p.Met1415Ile variant. Arrowheads show the heterozygous C > T mutation in the chromatograms

We encountered an 8-year-old female proband with SSc and a positive family history, which included a maternal grandfather who died of SSc and a maternal aunt with limited SSc (Fig. 2). The proband presented with severe Raynaud’s with dilated nailfold capillaries, capillary dropout, digital ulceration, digital scarring, and skin tightening over her face, arms, and legs. The patient displayed scleroderma facies with tightening of the skin around the eyes and lips with associated pallor. She did not show signs of organ fibrosis as shown by chest CT and echocardiogram. There were no signs of joint pain, swelling, stiffness, gastrointestinal symptoms, or rashes. A serological panel was performed for a spectrum of rheumatologic conditions, including ACA and ATA antibodies, which were all negative. These features meet the 2013 ACR/EULAR criteria for the classification of SSc [26]. Due to the very early onset of disease in this proband and the presence of a three-generation family history, we suspected a risk contribution from a rare variant of incomplete penetrance segregating in this family in an autosomal-dominant pattern. Consequently, we collected DNA specimens from five members of this family (Fig. 2) and we sequenced the exome in three individuals.

### Exome analysis

As described in the Methods, exomes underwent bioinformatic filtering to select protein-altering variants that fit the specified autosomal dominant inheritance model and which were rare, defined as less than 0.5 % for minor allele frequency. Variants meeting these criteria are itemized in Additional file 1: Table S1. The *NOTCH4* c.4245G > A:p.Met1415Ile variant has a Sorting Intolerance from Tolerance (SIFT) score of 0.02, which is predicted to be deleterious. Notably, the Exome Aggregation Consortium (http://exac.broadinstitute.org/variant/6-32168678-C-T) shows two heterozygous individuals out of 109,358 alleles for an allele frequency of 1.83 × 10^−5^, an extremely rare variant.

The next-generation sequencing results were validated by automated fluorescent Sanger sequencing (Fig. 2) and transmission in the predicted individuals was confirmed.

## Discussion

NOTCH4 is expressed almost exclusively in the endothelium and is thought to play an important role in the development of the vascular system. Considering the vascular abnormalities in this patient, the known contribution of vascular dysfunction in SSc, and the prior identification of a locus containing *NOTCH4* as a risk factor, we prioritized this variant.

Polymorphisms affecting expression of NOTCH4 have been implicated in a broad array of autoimmune diseases independent of their proximity to the HLA locus on chromosome 6p21. Here we have described a very rare amino acid substitution in a putative regulatory region of NOTCH4 segregating in a family with SSc/scleroderma.

We note that the mother of the proband appears disease-free despite carrying the exact same NOTCH4 p.Met1415Ile variant and without the expression of scleroderma. We are proposing that this mutation has less-than-100 % penetrance, which is frequently the case in autosomal dominant disease. Generally, the reason for this incomplete penetrance is not known. An alternative explanation would be polygenic inheritance, in which a phenotype arises from the additive interaction of a multitude of moderately impactful loci and displays a complex mode of inheritance. Systemic sclerosis/scleroderma ordinarily belongs to the polygenic category and it is associated with a multitude of SNPs which GWAS of SSc have shown to have effect sizes of OR < 2 outside the HLA region. This family appears to be a Mendelian phenocopy of the classic polygenically-inherited SSc because a very rare disease would be unlikely to occur with this three-generation history under a complex polygenic model of inheritance. Nevertheless, we cannot exclude the possibility that this variant is a false positive since we only have three affected carriers and the LOD score would not be expected to be genome-wide significant by classical linkage study criteria. This result is highly suggestive and is a starting point for functional studies that would focus on revealing the mechanism of NOTCH4 signaling.

As the least well-studied member of the NOTCH family, there is evidence that NOTCH4 functions in a manner unlike its paralogs and affects processes other than transcription. The results of James et al. suggest a unique post-translational processing of the receptor, since they found perinuclear localization of the protein and lack of proteolytic cleavage to form a heterodimer [27]. Of note, James et al. were unable to demonstrate autonomous signaling of the NOTCH4 receptor in HEK 293 cells, even when co-cultured with cells expressing the DSL family ligand [27].

The goal of functional analysis is further complicated by the fact that *Notch4* knockout mice do not have known phenotypic characteristics [28]. Double-knockout *Notch1*/*Notch4* mice show a more severe phenotype than *Notch1* knockout alone particularly with abnormal angiogenic vascular remodeling [28]. However, this family did not carry any rare variants in *NOTCH1*.

## Conclusion

No pathogenic mutations in the heterodimerization/NODP domain of NOTCH4 have been reported before. The genetic evidence from the family described here is supported by the known impact of NOTCH4 intracellular domain expression on the vascular endothelium in mouse models and the role of endothelium in the development of SSc. SNP-to-gene assignment in the analysis of complex traits by GWAS is a serious challenge for the genetics community. The segregation of this rare variant across three generations in a family argues that the variety of non-coding polymorphisms seen in autoimmune disease at the chromosome 6p21 genomic locus are affecting expression of the *NOTCH4* gene. We will seek to address the role of this mutation more definitively through the construction of a CRISPR knock-in mouse [29] bearing the variant so that its role in the vasculature and fibrosis can be assessed. Further evidence supporting the causal role of this variant should also be obtained in future studies focused on sequencing additional pedigrees for NOTCH4 mutations.

## Additional file

Additional file 1: Table S1. Table of rare coding variants segregating with the SSc/scleroderma phenotype. (XLS 27 kb)

## Abbreviations

ACA: Anti-centromere antibody
ACR/EULAR: American College of Rheumatology/European League Against Rheumatism
ANNOVAR: Annotation of variants
ATA: Anti-topoisomerase I antibody
BWA: Burrows-Wheeler aligner
DSL: Delta/serrate/lag
GATK: Genome analysis toolkit
GERP: Genomic evolutionary rate profiling
GWAS: Genome-wide association study
HD: Heterodimerization domain
HLA: Human leukocyte antigen
KING: Kinship-based inference for GWAS
LNR: Lin-12 Notch repeats
MHC: Major histocompatibility complex
NOD/NODP: NOTCH domain
NRR: Negative regulatory region
RBPJ-k: Recombination Signal Binding Protein for Immunoglobulin Kappa J Region
SIFT: Sorting intolerance from tolerance
SSc: Systemic sclerosis
WES: Whole exome sequencing
